# Enhanced Cropland SOM Prediction via LEW-DWT Fusion of Multi-Temporal Landsat 8 Images and Time-Series NDVI Features

**DOI:** 10.3390/s26031048

**Published:** 2026-02-05

**Authors:** Lixin Ning, Daocheng Li, Yingxin Xia, Erlong Xiao, Dongfeng Han, Jun Yan, Xiaoliang Dong

**Affiliations:** 1 School of Information Science and Engineering, Shandong Agricultural University, Taian 271018, China; ldc68825@163.com (D.L.); xinsinian2006@163.com (J.Y.); 2 Agricultural Big Data Research Center, Shandong Agricultural University, Taian 271018, China; dongxl@sdau.edu.cn; 3 Shandong Provincial Climate Center, Jinan 250031, China; hayecer@gmail.com

**Keywords:** soil organic matter, discrete wavelet transform, time-series NDVI features, convolutional neural network, digital soil mapping

## Abstract

Soil organic matter (SOM) is a key indicator of arable land quality and the global carbon cycle; accurate regional-scale SOM estimation is vitally significant for sustainable agricultural development and climate change research. This study evaluates a multisource data-fusion approach for improving cropland SOM prediction in Yucheng City, Shandong Province, China. We applied a Local Energy Weighted Discrete Wavelet Transform (LEW-DWT) to fuse multi-temporal Landsat 8 imagery (2014–2023). Quantitative analysis (e.g., Information Entropy and Average Gradient) demonstrated that LEW-DWT effectively preserved high-frequency spatial details and texture features of fragmented croplands better than traditional DWT and simple splicing methods. These were combined with 41 environmental predictors to construct composite Ev–Tn–Mm features (environmental variables, temporal NDVI features, and multi-temporal multispectral information). Random Forest (RF) and Convolutional Neural Network (CNN) models were trained and compared to assess the contribution of the fused data to SOM mapping. Key findings are: (1) Comparative analysis showed that the LEW-DWT fusion strategy achieved the lowest spectral distortion and highest spatial fidelity. Using the fused multitemporal dataset, the CNN attained the highest predictive performance for SOM (*R*^2^ = 0.49). (2) Using the Ev–Tn–Mm features, the CNN achieved *R*^2^ = 0.62, outperforming the RF model (*R*^2^ = 0.53). Despite the limited sample size, the optimized shallow CNN architecture effectively extracted local spatial features while mitigating overfitting. (3) Variable importance analysis based on the RF model reveals that mean soil moisture is the primary single variable influencing the SOM, (relative importance 15.22%), with the NDVI phase among time-series features (1.80%) and the SWIR1 band among fused multispectral bands (1.38%). (4) By category, soil moisture-related variables contributed 45.84% of total importance, followed by climatic factors. The proposed multisource fusion framework offers a practical solution for regional SOM digital monitoring and can support precision agriculture and soil carbon management.

## 1. Introduction

Soil organic matter (SOM) is a fundamental characteristic of soil [1] and is essential for sustaining soil ecological activities. Global soil is estimated to contain around 1.5 × 10^15^ kg of carbon in the form of SOM [2], and its dynamics directly impact the global carbon balance while also affecting agricultural production by influencing soil quality and fertility [3]. Accurate monitoring of SOM, as the primary source of nutrients essential for plant growth, serves as the scientific foundation for improving ecosystem management and is crucial for guaranteeing food security and attaining sustainable agricultural development.

Digital soil mapping (DSM) technology offers essential methodological and technological assistance for achieving high-precision monitoring of SOM. The DSM employs contemporary geographic information technology and statistical techniques to forecast and delineate the spatial distribution of soil characteristics. In contrast to conventional mapping that depends on expert expertise, it forecasts spatial variability using models and algorithms based on the association between soil data and environmental factors (e.g., climate, terrain, parent material, vegetation). In comparison to conventional soil mapping, DSM offers enhanced timeliness and precision [4].

In recent decades, satellite remote sensing technology has enabled the simultaneous monitoring of extensive portions of the Earth’s surface, offering dependable data support for the prediction of soil properties [5]. Various remote sensing sensors possess distinct observational perspectives and spectral response characteristics to their differing design principles and technical specifications [6], which directly influence the spatial matching accuracy of remote sensing image elements with actual feature samples, as well as the precision of spectral interpretation [7]. Research indicates that employing multitemporal remote sensing data enhances the ability to discern dynamic change patterns in surface spectral features, facilitating the extraction of stable feature information and so significantly mitigating the influence of nonsoil variables [8]. In the past decade, spatiotemporal fusion technology has achieved significant advancements [9], primarily through the amalgamation of remote sensing data with varying temporal, spatial, and spectral resolutions to produce a fused product that incorporates both high spatiotemporal resolution and extensive spectral information [10]. Research indicates that spatial fusion of multisource sensor data [11] and dynamic feature extraction from temporal data [8] can markedly enhance the predictive accuracy of soil properties. In recent years, numerous sophisticated fusion techniques have been developed, including the spatiotemporal adaptive reflectance fusion algorithm (STARFM) [12], enhanced spatiotemporal adaptive reflectance fusion algorithm (ESTARFM) [13], step-by-step multisensory fusion model [14], and fusion of phenological and spatiotemporal features [15], which offer enhanced technical support for remote sensing monitoring in agriculture. Traditional fusion techniques, like the intensity–hue–saturation transform [16] and high-pass filtering [17], can lead to distortion of the physical characteristics of images due to spectral resampling or spatial filtering processes. Although spatiotemporal fusion algorithms such as STARFM and ESTARFM have been widely used to generate synthetic high-resolution time series data, they are computationally intensive and primarily aimed at reconstructing missing images. It is crucial to preserve the original spatial texture and edge information in the context of SOM mapping of fragmented farmland. The wavelet transform, due to its multiresolution analysis properties and precise reconstruction ability, facilitates multilevel decomposition and fusion of remote sensing images across the entire frequency domain, enhancing spatial resolution while preserving spectral details. Discrete Wavelet Transform (DWT) has unique advantages in decomposing images into different frequency components, allowing for selective fusion of spatial details (high frequency) and spectral background (low frequency) [18]. However, traditional Discrete Wavelet Transform (DWT) fusion often employs fixed rules (e.g., maximum or mean) for high-frequency coefficients, which can lead to spectral distortion or the smoothing of spatial details, failing to capture complex agricultural micro-topography. To address this, this study introduces the Local Energy Weighted (LEW) strategy. The ability to enhance spatial features while effectively reducing spectral distortion by maximizing the preservation of local edge energy has been proven effective in soil attribute monitoring [19,20]. Since ‘energy’ directly reflects the richness of local image features (such as edges and textures), LEW-DWT can adaptively preserve information-rich details in high-frequency components while maintaining spectral consistency in low-frequency components. This approach significantly enhances the capture of cropland edges and textures, thereby providing more discriminative input features for high-precision SOM inversion. Meng et al. [21] conducted a study on typical cropland ecosystems in Northeast China and Inner Mongolia. They employed a region-weighted discrete wavelet transform approach that optimizes the weighting of spectral energy distribution across several regions. This approach facilitates the comprehensive integration of temporal features from 10 Landsat images (2009–2019), topography features from the SRTM-DEM, and hyperspectral features from GF-5 satellite data. Researchers have attained high-precision digital soil mapping by developing a temporal–spatial-spectrum (TSS) framework.

Conventionally, linear statistical models such as Partial Least Squares Regression (PLSR) have been widely employed for SOM prediction due to their computational simplicity. However, these methods are limited by their assumption of linearity, often failing to capture the complex, non-linear spatial dependencies between SOM and environmental variables, typically yielding moderate prediction accuracies [22]. Furthermore, studies relying on single-date remote sensing imagery face significant challenges; their predictive performance is highly sensitive to transient interference from soil moisture, crop residues, and atmospheric conditions at the time of acquisition, leading to poor model robustness and generalizability. Consequently, there is an urgent need for advanced methods that can model non-linear relationships and integrate multi-temporal information to overcome these accuracy bottlenecks. The random forest (RF), a fundamental technique in DSM, has been extensively utilized for predicting soil properties [23,24,25,26]. Numerous studies have shown that RF demonstrates enhanced prediction accuracy in DSM applications [26,27,28,29]. Deep learning, with their hierarchical feature learning mechanisms, has demonstrated distinct advantages in the analysis of remote sensing spectral features [30]. Convolutional neural network (CNN) exemplifies how sparse connectivity and weight sharing facilitate the automatic extraction of spatial correlation features from spectral data, thereby removing the subjectivity and empirical reliance characteristic of conventional manual feature design methods. By employing cascaded operations of various convolutional kernels, CNN can systematically extract multilevel features from raw multispectral and hyperspectral data, encompassing pixel-level spectral responses to regional-level land cover patterns. Research demonstrates that this feature learning paradigm can produce superior feature representations, markedly improving model predictive performance [31,32,33,34,35].

This work seeks to amalgamate multi-temporal multispectral data, temporal NDVI feature information, and environmental variable data. A high-precision model for predicting SOM is developed using machine learning and deep learning algorithms to spatially map SOM in cropland in Yucheng City. The specific objectives are: (1) to employ a novel fusion method, the Local Energy Weighted Discrete Wavelet Transform (LEW-DWT), to integrate long-term Landsat data to extract temporal information; (2) to develop a fusion of environmental variables, temporal NDVI feature, and multi-temporal multispectral information (Ev-Tn-Mm), and to evaluate the significance of the Ev-Tn-Mm fusion information for predicting SOM; (3) To evaluate the relative significance of multi-temporal multispectral information, timeseries NDVI feature information, and environmental variable data in forecasting SOM; (4) To generate a high-precision distribution map of SOM in arable land in Yucheng City.

## 2. Materials

### 2.1. Study Area

**Figure 1:**
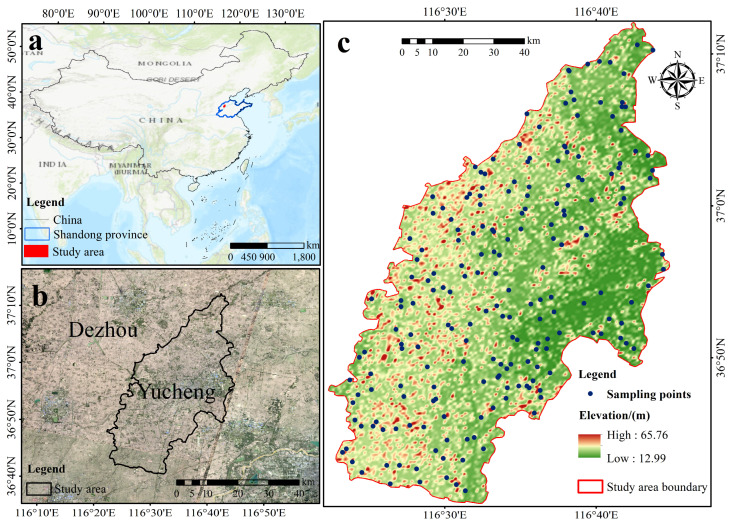
Study area and the locations of soil sample. (**a**) Geographical location map of the study area; (**b**) Satellite image of the study area; (**c**) Elevation and sampling point distribution map of the study area.

The study region is located in Yucheng city (Figure 1) in the alluvial plain of the Yellow River, northwestern Shandong Province, China (116°22′11″–116°45′00″ E, 36°41′36″–37°12′13″ N). It covers a total area of approximately 990.7 km^2^, with 99,000 hm^2^ of arable land. The region is characterized by a warm-temperate semi-humid monsoon climate, with concurrent rain and heat favorable for agriculture. The mean annual temperature is 14 °C, and the mean annual precipitation is 600 mm. The geomorphology is a typical Yellow River alluvial plain, and the soils are developed from Yellow River alluvium. According to the Chinese Soil Taxonomy, the dominant soil types are Fluvo-aquic soils and Saline-alkali soils, which generally correspond to Cambisols (or Fluvisols) and Solonchaks in the World Reference Base (WRB) system, respectively [36]. The soil texture is predominantly silt loam and sandy loam [37]. These soil characteristics, particularly the texture and hydro-physical properties associated with Fluvo-aquic soils, play a critical role in regulating the accumulation and decomposition dynamics of Soil Organic Matter (SOM) in this region. The agricultural system is characterized by an intensive double-cropping rotation of winter wheat and summer maize.

### 2.2. Soil Data

The SOM data in this study were derived from the Third National Land Resource Survey of Yucheng city. The sampling was conducted in 2020 in strict compliance with the Specifications for Cultivated Land Survey, Monitoring, and Evaluation. A conditioned Latin hypercube sampling (cLHS) method [38] was utilized for sample collection, considering parameters such as topography to ensure representation across varied landforms under agricultural land use types. Soil genetic horizons were identified at each sampling station, and surface soil samples (0–20 cm depth) were collected. A total of 198 topsoil sampling points were ultimately chosen. The measuring process strictly followed the Agricultural Industry Standard of the People’s Republic of China: Method for Determination of Soil Organic Matter. The 198 soil samples were air-dried, followed by the removal of plant roots and debris, sieving, grinding, and dry storage. The potassium dichromate volumetric technique was employed to ascertain the SOM concentration [39].

### 2.3. Covariate Data

The variables used for predicting the SOM included three major categories: multitemporal multispectral remote sensing imagery, time-series NDVI feature data, and environmental variables.

#### 2.3.1. Multi-Temporal Multispectral Remote Sensing Imagery

Seven Landsat 8 OLI images from 2014 to 2023, as listed in Table 1, were obtained from the Geospatial Data Cloud platform (https://www.gscloud.cn) to capture bare soil conditions (cloud cover < 5%). The justification for this time selection was threefold: (1) Prior research indicates that SOM generally exhibits negligible fluctuations over a decade [40]; (2) cloud interference hindered the collection of suitable bare soil images in 2016, 2021, and 2022; and (3) the soil sampling initiative for this study occurred during the bare soil interval in 2020. All images possessed a spatial resolution of 30 m.

The Landsat 8 OLI images underwent rigorous preparation. Initially, radiometric calibration was executed using the radiation calibration module. Thereafter, atmospheric adjustment was performed using the FLAASH module. Ultimately, all images were spatially cropped utilizing the vector border of the study region.

#### 2.3.2. Time-Series NDVI Feature Data

The NDVI is a prevalent remote sensing metric for monitoring vegetation, accurately representing coverage, biomass, and growth conditions. Due to the significant association between vegetation dynamics and SOM variation [41], the NDVI has been frequently employed as a principal proxy indicator for evaluating the geographical distribution and temporal fluctuations in SOM [42]. The NDVI is calculated as seen in Equation (1).

(1) $$NDVI=\frac{NIR-RED}{NIR+RED}$$

This study obtained MODIS remote sensing images data from 2014 to 2023, spanning a decade, to develop a time-series dataset that encompasses multiple complete cycles, ensuring the reliability and validity of the research. The temporal resolution of the images was 16 days, with a spatial resolution of 250 m. An average of 23 photos was acquired annually, culminating in a total of 230 photographs. The time-series NDVI dataset was subsequently computed and obtained. Detailed information is provided in Table 2.

#### 2.3.3. Environmental Variables

The environmental variables used for SOM prediction include seven categories: climate, parent material, topography, vegetation, surface thermal conditions, soil, and human activities. Specific variable information is shown in Table 3. The soil moisture data (2003–2018) was obtained from the dataset generated by Meng et al. [43]. This dataset was developed by fusing and downscaling passive microwave remote sensing data derived from AMSR-E, AMSR2, and SMOS sensors. We utilized the multi-year average soil moisture to represent the long-term stable spatial distribution of soil water content, which serves as a key climatic background factor influencing SOM accumulation, rather than instantaneous moisture conditions.

### 2.4. Data Preprocessing

All environmental variable data, characterized by differing resolutions, underwent preprocessing including projection transformation, geometric correction, image cropping, and resampling, resulting in a consistent spatial resolution of 30 m. To mitigate the uncertainty caused by scale conversion and avoid blocky artifacts, different resampling methods were evaluated and applied based on data types. Specifically, Cubic Convolution interpolation was employed for continuous coarse-resolution variables (e.g., MODIS NDVI and Soil Moisture) to generate smooth spatial gradients consistent with the 30 m grid, while Nearest Neighbor resampling was used for categorical data (e.g., Soil Type). This dataset amalgamates environmental variables, multi-temporal multispectral remote sensing imagery, and NDVI time-series attributes.

## 3. Methods

**Figure 2:**
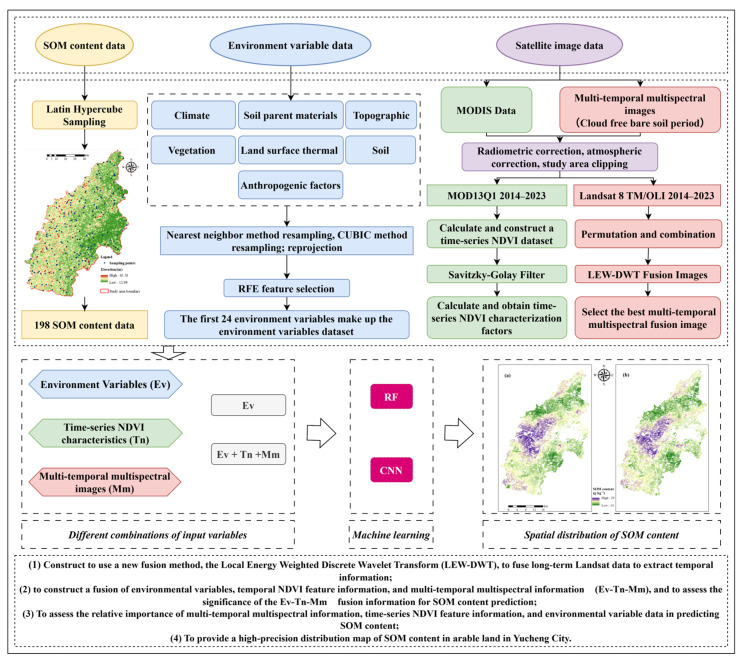
Methodological framework for SOM prediction via LEW-DWT fusion. (**a**) Distribution of SOM prediction results under Ev conditions. (**b**) Distribution of SOM prediction results under Ev-Tn-Mm conditions.

This study seeks to develop a high-precision model for predicting SOM at a regional scale. For the selection of predictor factors, we thoroughly integrated environmental variables, time-series NDVI features, and multitemporal multispectral data (Figure 2). The original time-series NDVI dataset was subjected to Savitzky–Golay (S-G) filtering [47] to facilitate pixel-by-pixel smoothing, fitting, and reconstruction, from which representative time-series NDVI features were derived. The LEW-DWT method was applied to fuse multitemporal multispectral data, carefully evaluating the impacts of various features permutations and combinations to identify the ideal multitemporal multispectral dataset. Regarding model construction, we utilized RF and CNN methodologies to identify the optimum model for predicting SOM. Furthermore, by computing variable significance metrics, the impact of environmental features on the prediction of SOM was objectively evaluated. Utilizing the optimal model, we generated a spatial distribution map of the SOM for arable land in Yucheng city.

### 3.1. Extraction of Time-Series NDVI

The frequency-domain features of the time-series were extracted from the NDVI dataset. This study employed S-G filtering (Equation (2)) on the NDVI time-series data. This approach utilizes locally weighted least squares to ascertain polynomial fitting coefficients for moving-window weighted averaging, thus facilitating pixel-by-pixel smoothing and reconstruction of the time-series data. The reconstructed NDVI minimum (N_Min), maximum (N_Max), standard deviation (N_Std), and additional statistical features were extracted pixel by pixel. The vegetation growing season length (N_GSL) was determined through the thresholding method. The Fourier transform was applied to the smoothed time-series data post-S-G filtering, and the first harmonic amplitude was derived from the Fourier transform calculations. The amplitude (N_Amplitude) and phase (N_Phase) characteristics were derived from the spoke angle of the fundamental harmonic.

(2) $$\underset{a_{0},a_{1},\cdots a_{k}}{\min}\sum_{i=-m}^{m}{\left(y_{i}-(a_{0}+a_{1}x_{i}+a_{2}x_{i}^{2}+\cdots +a_{k}x_{i}^{k})\right)}^{2}$$

where *x_i_* is the position of the relative center point in the window, *y_i_* is the corresponding NDVI observation, *k* is the polynomial order, and 2*m* + 1 is the window_length.

### 3.2. Extraction of Multitemporal Multispectral Data

Seven Landsat 8 images were utilized for SOM prediction (Table 4), evaluating changes in SOM prediction performance by progressively increasing the number of images, with the objective of investigating the relationship between SOM prediction efficacy and multi-temporal multispectral data.

The multispectral image from 2020 was utilized as the baseline image, as the soil samples were gathered in 2020. When a single image was evaluated, the 2020 image was chosen as the input variable, designated as MMI_1_; when two images were assessed, the 2020 image was combined with one of the other six images, and the resultant fused image was labeled as MMI_2_. According to the combination formula (Equation (3)), the total number of combinations for MMI_2_ was 6. Irrespective of the quantity of multitemporal multispectral images, ranging from 1 to 7, all input variables encompass 7 bands.

(3) $$C\left(n,k\right)=\frac{n!}{k!\left(n-k\right)!}$$

where *n* denotes the total number of elements (except for 2020, where n = 6), and *k* denotes the number of elements to be selected.

### 3.3. Fusion of Multitemporal Multispectral Data

**Figure 3:**
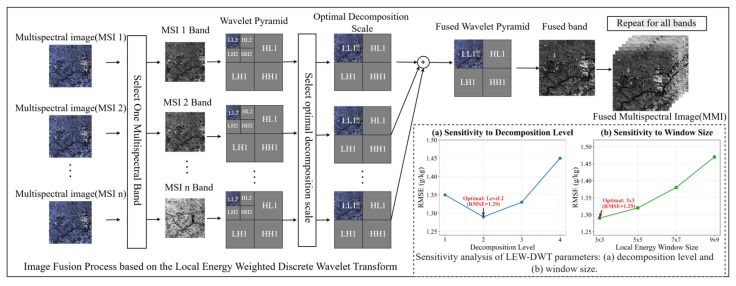
Fusing multispectral images using the LEW-DWT method and sensitivity analysis of LEW-DWT parameters: (**a**) decomposition level and (**b**) window size.

The Discrete Wavelet Transform (DWT) approach entails the assessment and computation of image energy, image decomposition, and image reconstruction [21]. This research employs the LEW-DWT fusion technique to achieve the fusion of multitemporal multispectral information. The specific process is shown in Figure 3.

The computation of image energy initially requires the acquisition and conversion of data values from each band. According to the principle of discrete sampling, the pixel values of each band are normalized and transformed into floating-point matrices to satisfy the criteria for numerical operations. The energy value of the corresponding band is subsequently derived by computing the sum of the squares of each matrix member (Equation (4)). This metric functions as a significant statistical attribute, accurately delineating the information density of the band [48]. In image fusion applications, an adaptive weight allocation approach grounded in energy values [49] can emphasize the preservation of spectral characteristics with greater informational richness, hence optimizing critical metrics such as information entropy and spatial resolution in the fusion outcomes.

Within the wavelet transform paradigm, image decomposition facilitates feature extraction via multiscale analysis. The original image is decomposed using a wavelet transform to produce four feature components: the low-frequency component (LL) denotes the overall approximation characteristics of the image, while the three high-frequency components (HL, LH, HH) encapsulate spatial detail features in distinct orientations (horizontal, vertical, and diagonal). The decomposition process is performed iteratively in a cascading fashion, with the maximum decomposition scale dictated by the signal length and the wavelet basis function. This research utilizes the Haar wavelet basis function for secondary decomposition. To determine the optimal decomposition level, we conducted sensitivity tests on the decomposition of layers 1 to 4 (as shown in Figure 3). The results indicate that the first level decomposition preserves insufficient spatial details, while the third level and above decomposition introduces excessive high-frequency noise. In contrast, Level 2 decomposition achieved the best balance between preserving multispectral spectral information and enhancing spatial texture features. Therefore, this study ultimately chose to perform Level 2 decomposition. Subsequent to normalizing the energy of each band (Equation (5)), an energy-weighted fusion algorithm efficiently integrates the wavelet coefficients of multisource pictures (Equation (6)). This approach computes weighted fusion coefficients for each band, finally producing a fusion result that includes optimized approximation and detail coefficients. This process efficiently preserves the most informative feature components from each source image.

The image reconstruction procedure utilizes an inverse wavelet transform to convert the fused multiscale coefficients into a spatial-domain image. This procedure employs a fusion rule predicated on local energy [50]. Specifically, the calculation of local energy is based on a 3 × 3 sliding window. Parameter sensitivity analysis shows (as shown in Figure 3) that a small window of 3 × 3 can most sensitively capture small texture changes and edge features on the surface of cultivated land; as the window size increases (such as 5 × 5 or 7 × 7), local details are excessively smoothed, resulting in a decrease in the spatial resolution of the reconstructed image and the final SOM prediction accuracy. Therefore, at each spatial position, the feature component exhibiting more local energy is given precedence. In the selection of low-frequency subbands, the approximation component containing the most information is preserved, whereas in the processing of high-frequency subbands, the component with the most significant detailed features is chosen. This selective fusion technique effectively guarantees that the reconstructed image retains its overall structure while maximizing the preservation of the most significant characteristics from each source image.

(4) $$E=\sum (x_{i}^{2})$$

(5) $$W_{i}=\frac{E_{i}}{\sum_{j=-1}^{N}E_{j}}$$

(6) $$C_{fused}=\sum_{i=1}^{N}W_{i}\cdot C_{i}$$

*x_i_* denotes the pixel value, *E_i_* is the energy of the ith image, and *N* is the number of images involved in the fusion. *C_i_* is the wavelet coefficient of the i image, and *W_i_* is its corresponding energy weight.

### 3.4. Feature Selection and Experimental Categorization

In model construction, numerous covariances among environmental variables diminish model interpretability and elevate the risk of overfitting. This work employed recursive feature elimination (RFE) to evaluate environmental factors and mitigate the covariance issue [51]. Prior to modeling, the RFE algorithm was executed 10 times to determine the average selection frequency of each variable, resulting in the selection of the top 24 variables with optimal performance as the final predictive factors for subsequent modeling study.

This work designed two sets of variable combinations for comparative analysis to systematically evaluate the efficacy of LEW-DWT-fused approach in predicting cropland SOM (Table 5).

### 3.5. Model Selection

A multitude of predictive methods for DSM are already accessible. This study used two models, RF and CNN, for comparative analysis.

#### 3.5.1. RF

RF is an integrated learning approach based on decision trees, improves prediction accuracy and model robustness by constructing multiple decision trees and subsequent voting or averaging. n_estimators and max_depth are the core parameters in the RF [1,52]. The ideal parameters are determined using the GridSearchCV, with n_estimator (100, 150, 200, 250, 300, 350), and max_depth (3, 4, 5, 6, 7, 8). The results indicate that the ideal n_estimator is 250 and the max_depth is 6.

#### 3.5.2. CNN

**Figure 4:**
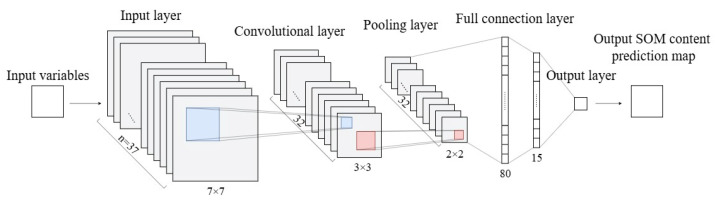
CNN structure diagram.

CNN is a deep learning architecture designed for the analysis of grid-structured data [53]. The typical design comprises input layer, convolutional layer, pooling layer, dropout layer, fully connected layer, and output layer (Figure 4). The input layer represents image data as a three-dimensional array (channels × height × width). The convolution layer, as the core component of CNN, executes sliding convolution operations on the input data using a collection of learnable filters to efficiently extract local spatial characteristics. Nonlinear activation functions are implemented following each convolution operation to enhance the model’s expressive capacity. The subsequently linked pooling layer markedly decreases the dimensions of the feature map by downsampling procedures, which reduces the number of parameters while preserving essential features, thus effectively mitigating the overfitting phenomenon. At the end of the network, the feature maps resulting after multiple convolutions and pooling are transformed into one-dimensional vectors enabling sophisticated feature integration via the fully connected layer. The final output layer employs an activation function appropriate for the task to output the probability distribution of the predicted outcome.

The model training utilized a stochastic gradient descent technique founded on the Adam optimizer, with hyperparameter optimization executed by the grid search method [54]. To improve model performance, several measures have been incorporated into the CNN architecture: the mean squared error (MSE) has been selected as the loss function; dropout layers have been integrated to alleviate overfitting resulting from intricate model structures [55,56]; and ReLU activation functions have been employed to facilitate nonlinear transformations in neurons [57]. In the SOM prediction, utilizing a 7 × 7 window with varying channel numbers corresponding to the input variables to encapsulate both sample and neighborhood information. A 3 × 3 convolutional window was utilized to efficiently extract spatial feature information from samples and their adjacent regions. To ensure the robustness of the network architecture and identify optimal hyperparameters, we conducted a parameter sensitivity analysis based on the Grid Search method. The search space included: the number of convolutional layers (2, 3, 4), filter sizes (2 × 2, 3 × 3, 5 × 5, 7 × 7), and Dropout rates (0.1, 0.2, 0.5). Empirical results indicated that, given the fragmented nature of the cropland landscape, smaller filters (3 × 3) outperformed larger ones in capturing local texture details. Furthermore, considering the limited sample size, a deeper network (>4 layers) led to overfitting, while a shallower network (2 layers) lacked sufficient feature extraction capacity. Consequently, the finalized architecture (Table 6) with three convolutional layers and 3 × 3 filters represented the optimal combination for balancing prediction accuracy and model generalization on this dataset.

### 3.6. Modeling and Evaluation

In this study, RF and CNN models were constructed for SOM prediction. Given the limited sample size (*N* = 198) and the data-intensive nature of the CNN model, we adopted a 10-fold cross-validation (CV) strategy instead of using a fixed independent validation set. This approach ensures that all samples are iteratively used for both training and validation, thereby maximizing data utilization while providing a robust estimate of mapping accuracy and model stability [58]. Consequently, the optimal model configurations were determined through this CV process to ensure the reliability of the performance evaluation [39].

Three assessment metrics—mean absolute error (MAE), root mean square error (RMSE), and coefficient of determination (*R*^2^)—were computed to assess the model’s accuracy.

(7) $$MAE=\frac{1}{n}\sum_{j=1}^{n}\left|\left(P_{n}-Q_{n}\right)\right|$$

(8) $$RMSE={\left[\frac{1}{n}\sum_{j=-1}^{n}{\left(P_{n}-Q_{n}\right)}^{2}\right]}^{0.5}$$

(9) $$R^{2}=1-\frac{\sum_{j=1}^{n}{\left(P_{n}-Q_{n}\right)}^{2}}{\sum_{j=1}^{n}{\left(P_{n}-\bar{Q_{n}}\right)}^{2}}$$

where *n* is the number of predictions, *P_n_* is the observed SOM value at *n* points, and $\bar{Q_{n}}$ is the predicted SOM value at *n* points, averaged over *Q_n_*.

## 4. Results

### 4.1. Statistical Analysis of SOM Samples

This research performed a systematic statistical analysis of the SOM in 198 soil samples (Table 7). The results indicated that the SOM in the study region varied from 14.73 to 27.65 g/kg. The coefficient of variation (CV) was 12%, showing that the SOM displayed moderate to weak geographic variability. This variability pattern indicates that the general distribution of SOM in the research area is reasonably consistent, however discernible spatial variability persists. The modest variability guarantees sample representativeness while supplying the essential data basis for developing a spatial prediction model for SOM.

### 4.2. Analysis of Fused Multitemporal Multispectral Images

**Figure 5:**
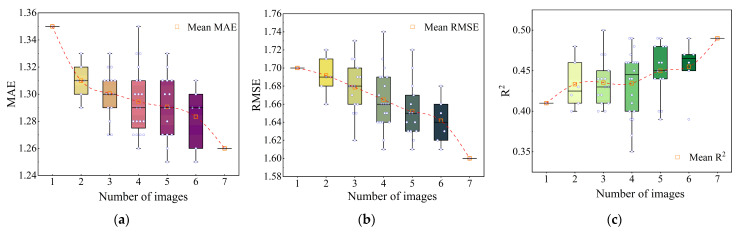
Comparison of SOM inversion accuracy under different numbers of image inputs. (**a**) Mean MAEs, (**b**) mean RMSEs, (**c**) mean *R*^2^ values. The red dashed lines represent the trends of mean MAEs, mean RMSEs, and mean *R*^2^ respectively.

Table 8 presents the maximum accuracies of different numbers of multitemporal multispectral images and their corresponding fused images captured at different intervals. Specifically, when utilizing a singular year of image input (2020), the CNN model exhibits an accuracy *R*^2^ of 0.41, surpassing the *R*^2^ = 0.3 for RF; thus, the following modeling and analysis of multitemporal multispectral data is conducted using CNN. The findings indicate that the predictive accuracy enhances as the number of input images increases. The MMI_7_ combination model, encompassing all seven images, exhibited the highest performance, achieving a *R*^2^ of 0.49, signifying the most robust fit. Conversely, the MMI_1_ combination, which utilizes a solitary picture, performs the worst, with MAE = 1.35, RMSE = 1.70, and *R*^2^ = 0.41. Figure 5 illustrates the correlation between the accuracy of SOM inversion and the number of images. The findings show that a judicious augmentation in the number of images can effectively enhance the performance of the prediction model. Consequently, the MMI_7_ combination is regarded as the ideal image amalgamation for subsequent modeling analysis.

To explicitly evaluate the quality of the fused images, we employed three quantitative metrics: Spectral Angle Mapper (SAM), Information Entropy (IE), and Average Gradient (AG) [59]. SAM measures the spectral distortion compared to the baseline image (2020), where a smaller value indicates higher spectral fidelity. IE reflects the richness of spatial information, and AG characterizes the clarity of textures and edges; for both, higher values are desirable. We compared the fusion performance of the proposed LEW-DWT against the Standard DWT. The quantitative results are presented in Table 9. The LEW-DWT method achieved a lower SAM value (0.038) compared to Standard DWT (0.056), indicating superior capability in preserving the original spectral characteristics of the soil surfaces. Furthermore, LEW-DWT exhibited significantly higher values in both IE (5.65) and AG (4.92). This confirms that the local energy-weighted strategy effectively integrates complementary information from multi-temporal phases and enhances high-frequency spatial details (e.g., field boundaries and texture), which are critical for differentiating SOM levels in heterogeneous croplands.

### 4.3. Time-Series NDVI Characterization

#### 4.3.1. Characterization

**Figure 6:**
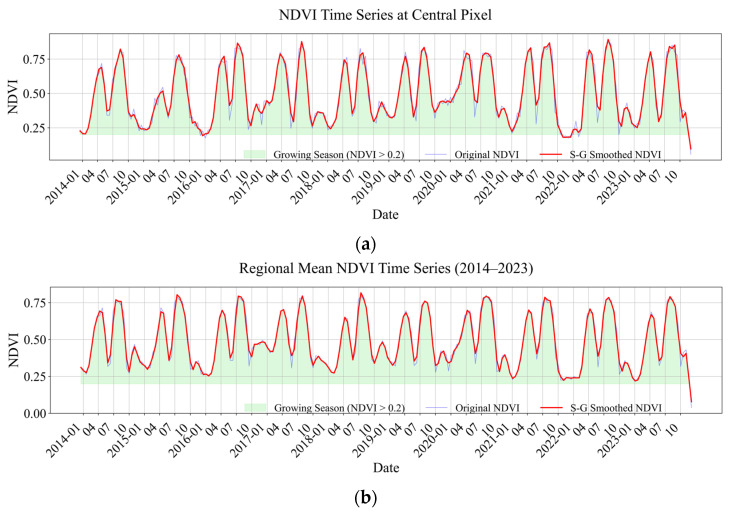
(**a**) S-G fitting curves representing time-series NDVI features at the central pixel; (**b**) S-G fitting curves representing regional mean time-series NDVI features.

Figure 6 illustrates the variation curves derived from the fitting and smoothing of the NDVI time-series dataset from 2014 to 2023 using the S-G filtering method. An examination of the two time-series plots revealed that the S-G technique significantly enhanced the quality of the NDVI time-series data. At the pixel level, the original NDVI data exhibit considerable seasonal variability and random noise; however, S-G filtering effectively mitigates outliers (such as the anomalously low values at the onset of the growing season) while distinctly preserving essential climatic characteristics. The regionally averaged NDVI series demonstrated that the approach could eradicate systematic mistakes (e.g., summer sensor bias) and precisely reflect regional vegetation cycles. Both the unprocessed NDVI and the filtered NDVI exhibit clear seasonal regularity. At the pixel level, the maximum value of the S-G-filtered NDVI remained consistent at 0.7–0.8, while the minimum value was sustained at 0.2–0.3. In contrast, the maximum value of the S-G-filtered NDVI for the regional average ranged from 0.6 to 0.7, with the minimum value between 0.1 and 0.2. The NDVI curves at the pixel scale exhibited greater variability, likely attributable to the enhanced development of plants resulting from the elevated levels of local SOM. Furthermore, the time-series NDVI features at varying spatial scales exhibited substantial differences: the pixel-scale curve demonstrated heightened sensitivity to fluctuations induced by field management practices, while the regional curve more accurately reflected the overarching climatic pattern. The disparities indicate that time series indicators, including N_GSL, N_Amplitude, and N_Phase, must be thoroughly evaluated in the extraction of vegetation characteristics to adequately delineate the impact of SOM on vegetation growth dynamics.

#### 4.3.2. Correlation Analysis of Time-Series NDVI Features

**Figure 7:**
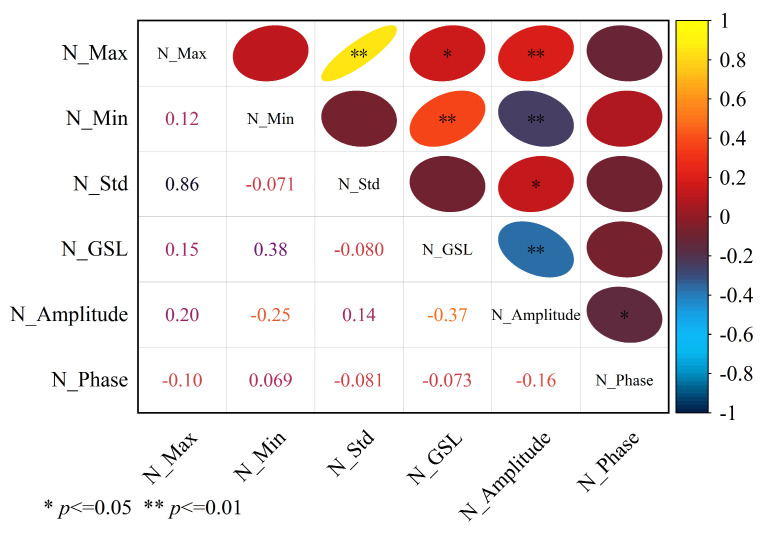
Correlation analysis between time-series NDVI features. The size of the circle represents the absolute value of the correlation coefficient (the larger the circle, the stronger the correlation).

To evaluate the interconnections among the time-series NDVI features, we performed a correlation analysis (Figure 7), with values ranging from −0.37 to −0.86, indicating varying degrees of correlation; the reliability of these correlations was subsequently verified using a significance test. The results indicated strong relationships between some feature pairs, although the strength of the correlations among the variables varied considerably.

### 4.4. Analysis of Variable Importance Based on RF

**Figure 8:**
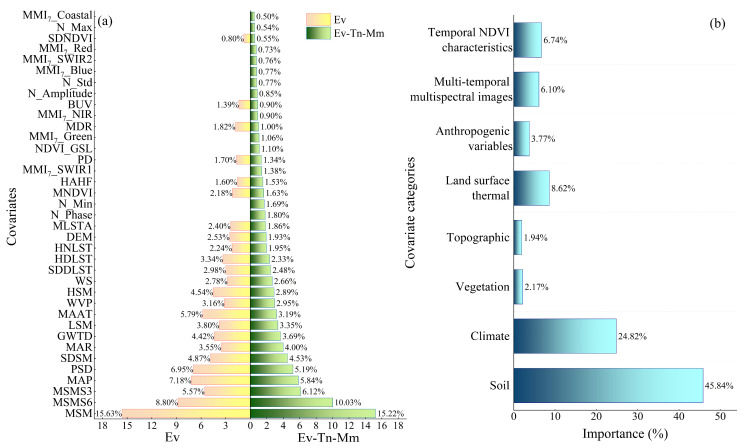
(**a**) Indicates the importance of input variables. (**b**) Indicates the importance of different categories of variables.

Given the “black box” nature of CNN models, which makes direct interpretation of variable contributions challenging, we utilized the interpretable Random Forest (RF) model to quantify the relative importance of different environmental covariates. This analysis aims to identify the primary environmental drivers governing the spatial distribution of SOM in the study area based on pixel-level statistical correlations, rather than to directly map the internal weights of the CNN. Figure 8 shows the relative importance of each variable. The research indicated that the MSM was the primary predictor for SOM prediction, with a relative value of 15.22%. Subsequently, MSMS6 and MSMS3 ensued. MSM, MSMS6, MSMS3, MAP, and PSD emerged as the five most important input factors, all of which had a strong correlation with SOM.

The analysis of the predictive importance of time-series NDVI features for SOM prediction indicated that N_Phase was the most important feature, contributing 1.80%, underscoring the vital role of vegetation’s key seasonal period as an indicator of SOM dynamics; N_Min (1.69%) and N_GSL (1.10%) followed as the next most important features, illustrating the considerable impact of vegetation’s extreme growth status on SOM. Secondary variables, including N_Amplitude (0.85%), N_Std (0.77%), and N_Mmax (0.54%), delineate alterations in vegetation growth dynamics. This ranking demonstrates that the characteristics of vegetation phenology significantly explain SOM variation, and the key role of N_Phase suggests a phenological dependence in the vegetation–soil material cycle, thereby informing the selection of variables to enhance the SOM spatial prediction.

The investigation indicated variations in the explanatory efficacy of the remote sensing image bands for predicting SOM following the integration of the 7-year time-series data. The MMI_7__SWIR1 band emerges as the most predictive variable, possessing an importance share of 1.38%, likely due to its heightened sensitivity to the spectral response of SOM. The MMI_7__Green band follows as the second most significant band at 1.06%, and together, they represent the fundamental indicators of multispectral prediction. The importance of the residual bands is as follows: MMI_7__NIR (0.9%), MMI_7__Blue (0.77%), MMI_7__SWIR2 (0.76%), MMI_7__Red (0.73%), and MMI_7__Coastal (0.5%). These bands exhibit a gradient distribution characteristic, illustrating the variations in the capacity of distinct spectral intervals to signify the SOM. Notably, the middle band (green~SWIR1) demonstrates a significantly greater predictive value. The results offer an ideal band combination technique for SOM remote sensing inversion and confirm the significant impact of long-term time-series data fusion on enhancing the predictive of soil parameters.

This work comprehensively measured the contribution of several variable categories to the prediction of SOM (Figure 8b), based on the results of the individual variable importance analysis (Figure 8a). The results indicated considerable disparities in the contribution of each environmental category to prediction accuracy, demonstrating clear category specificity. The soil category parameters accounted for 45.84%, underscoring the primary influence of soil background on SOM distribution. The climate parameters placed second, contributing 24.82%, mostly indicating the regulating influence of hydrothermal conditions on the decomposition–accumulation process of SOM. The contribution rate of the land surface thermal category variables was 8.62%, placing it third, indicating the impact of the surface energy balance on soil biochemical activities. The time_series NDVI factors (6.74%) and multitemporal multispectral data (6.10%), despite their relatively low percentages, furnished the model with additional information that standard environmental variables could not substitute, owing to their distinctive capacity for dynamic monitoring. The very minor influences of topography (3.21%) and vegetation type (2.67%) may be attributable to the unique geo-environmental circumstances of the area.

### 4.5. Prediction Accuracy

This work systematically elucidates the performance of the RF and CNN in SOM prediction across two combinations of input variables (Table 10). The findings indicate that the CNN model possesses considerable advantages in terms of predictive accuracy and stability. While the RF generally enhances performance, the extent of increase is somewhat constrained. In the assessment of RF, expanding the input data from the singular variable Ev to the Ev-Tn-Mm combination results in an increase in R^2^ from 0.47 to 0.53, reflecting a relative enhancement of 12.8% in the explanatory power. The MAE diminishes from 1.28 to 1.18, reflecting a reduction of 7.8%; the RMSE lowers from 1.59 to 1.52, indicating a decline of 4.4%. The RMSE/MAE ratio of RF consistently averages about 1.3 across all test datasets. This outcome suggests that the distribution of prediction errors is comparatively steady; nevertheless, it also indicates that the model exhibits a relatively low sensitivity to enhancements in data quality. Conversely, the CNN demonstrates more pronounced performance enhancement attributes. The R^2^ rises markedly from 0.54 in the Ev condition to 0.62 in the Ev-Tn-Mm condition, reflecting a relative increase of 14.8%. Regarding error management, the CNN exhibits superior performance: the MAE diminishes by 19.5%, and the RMSE reduces by 14%. A comprehensive examination indicates that the RMSE/MAE ratio of CNN rises from 1.33 for Ev to 1.42 for Ev-Tn-Mm, suggesting that as data quality improves, the model sustains overall predictive accuracy while concurrently augmenting its capacity to manage extreme values. The comparative research reveals substantial differences between the two models across three critical performance metrics. In terms of explanatory power, the R^2^ advantage increases from 12.8% of RF to 14.8% of CNN, demonstrating an accelerating improvement trend, whereas the RF exhibits a more gradual enhancement. Secondly, regarding error control, under the Ev-Tn-Mm condition, the CNN’s MAE and RMSE are 22.9% and 15.1% lower than those of RF, respectively, and this advantage continues to increase with data optimization. Regarding predictive stability, the error dispersion (RMSE/MAE) of CNN improves with enhanced data quality. The disparities mostly arise from the architectural benefits of the CNN model for automatic feature extraction and the modeling of nonlinear relationships, particularly when dealing with high-dimension data, as these advantages are more evident. Furthermore, to assess the practical utility of the model, we compared the prediction error against the natural variability of the field data. The Standard Deviation (SD) of the observed SOM was 2.42 g/kg (CV = 12%), which represents the baseline error if a simple mean value were used for mapping. The CNN model achieved an RMSE of 1.29 g/kg, corresponding to a relative RMSE of approximately 6.4%. Since the model’s error (6.4%) is substantially lower than the natural variation (12%), and the RMSE (1.29) is nearly half the SD (2.42), the proposed method demonstrates significant predictive value beyond a simple field average.

To further validate the effectiveness of the proposed LEW-DWT fusion strategy, we compared it with two control methods: (1) Simple Splicing, which directly concatenates resampled multi-source bands as input features; and (2) Traditional DWT, which utilizes the discrete wavelet transform but employs a simple ‘mean rule’ for fusing coefficients without local energy weighting. The comparison results (shown in Table 11) indicate a clear hierarchy in performance. The Simple Splicing method yielded the lowest accuracy (RMSE = 1.45, R^2^ = 0.53), likely due to its inability to extract multi-scale spatial features. The Traditional DWT improved performance (RMSE = 1.37, R^2^ = 0.57) by incorporating frequency domain information. However, the proposed LEW-DWT achieved the highest accuracy (RMSE = 1.29, R^2^ = 0.62). Quantitatively, LEW-DWT reduced the RMSE by approximately 5.8% and 11.0% compared to Traditional DWT and Simple Splicing, respectively. This demonstrates that the local energy-weighted rule effectively preserves critical spatial details and texture information from the high-resolution imagery, which are essential for accurate field-scale SOM mapping.

### 4.6. Spatial Distribution and Uncertainty of SOM Prediction

**Figure 9:**
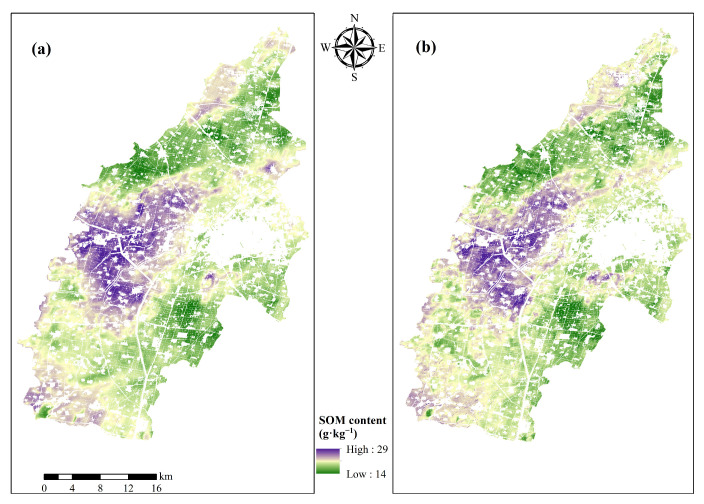
Distribution of the SOM predicted results under Ev (**a**) and Ev-Tn-Mm (**b**) conditions.

**Figure 10:**
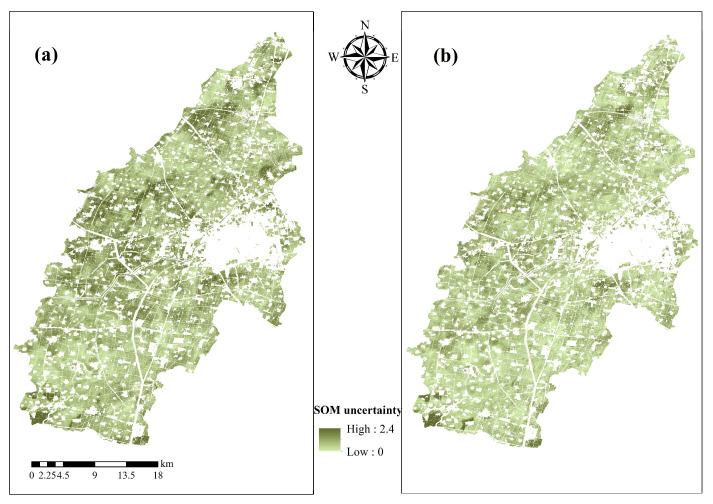
SOM uncertainty distributions under Ev (**a**) and Ev-Tn-Mm (**b**) conditions.

This study examines the impact of various combinations of input variables on the prediction of SOM by comparing the accuracy of prediction results (Figure 9) and uncertainty distributions (Figure 10) for two distinct variable combinations, utilizing the most effective CNN model.

The spatial distribution revealed that SOM in the area demonstrated considerable spatial variation. The predicted range of SOM for solely environmental variables (Figure 9a) was 15.03 to 25.53 g/kg, whereas the predicted range for Ev-Tn-Mn conditions (Figure 9b) expanded to 14.49 to 28.62 g/kg. To facilitate a direct comparison of spatial patterns between the two models, the color scale in Figure 8 is standardized to a unified range of 14–29 g/kg. The spatial distribution of SOM revealed that high concentrations were primarily situated in the central-western and southwestern regions of the research area, whereas low concentrations were predominantly found in the northern, eastern, and southeastern regions. The model utilizing solely environmental factors represented the general spatial distribution of SOM, whereas it lacked precision in precise characterisation. The comprehensive model integrating all variables not only markedly enhanced the predictive accuracy of intensive farmland utilization areas but also more precisely delineated the microtopography-induced variations in SOM, thereby improving the model’s adaptability to seasonal fluctuations in soil characteristics.

The uncertainty analysis of SOM prediction with various variables (Figure 10) indicates that the uncertainty incorporating solely environmental variables (Figure 10a) spans from 0.03 to 2.35, while the uncertainty of the Ev-Tn-Mn model (Figure 10b) is diminished to 0.07 to 1.90. Visualized under a unified color scale (e.g., 0–2.40), Figure 10b exhibits noticeably lower values across the region compared to Figure 10a. The comparison demonstrates that the introduction of time-series NDVI characteristics and multitemporal multispectral data markedly diminishes the uncertainty of model predictions, hence affirming the critical role of multisource data fusion in enhancing the stability of SOM predictions.

## 5. Discussion

### 5.1. Relative Importance of Variables

The importance ranking based on RF primarily reflects the statistical correlation between pixel-scale environmental factors and SOM. While this cannot be entirely equated to the feature learning mechanism of CNN, the dominance of key variables (e.g., soil moisture) provides a solid ecological foundation for prediction. The limitation of RF lies in its inability to quantify the contribution of spatial neighborhood features, which is precisely where CNN excels. CNN extracts spatial textures and contextual information of these key variables (e.g., MSM) through convolutional layers, thereby further improving prediction accuracy (R^2^ increasing from 0.53 to 0.62) over RF. Thus, RF reveals “which variables are important,” while CNN achieves superior mapping performance by learning the “spatial patterns” of these variables.

#### 5.1.1. Relative Importance of Environmental Features

This study carefully assessed the relative significance of several predictors, revealing that the contributions of different environmental variables differed. Climate affects the geographical variation in SOM [60], a crucial element in soil formation. Research indicates that precipitation and temperature are two principal climatic variables affecting the geographical variability of SOM, as corroborated by other research in analogous places [1,3,61]. In the comprehensive model accounting for all variables, the relative significance of MAP and MAAT was placed 4th and 10th, respectively. These climatic conditions impact SOM by affecting carbon input and decomposition processes. Precipitation is critical for net primary productivity, which subsequently influences soil carbon input. Increased humidity promotes the weathering of parent rocks and the stability of soil carbon on mineral surfaces [62], hence aiding in the reduction in SOM decomposition [63]. Conversely, temperature impacts the rate of microbial decomposition of SOM [64], as it regulates the metabolic activity of microorganisms and enzyme activity, hence affecting the decomposition process of SOM.

LST indirectly influences SOM concentration by impacting microbial activity and the rate of organic matter decomposition [65]. The heightened occurrence of warm nights resulting from increasing LST considerably intensified the depletion of SOM, while the decrease in cold days somewhat alleviated the adverse impact of elevated temperatures. This action indirectly influences SOM dynamics by modifying soil moisture evaporation, microbial metabolic activity, and plant root exudate inputs [66]. Soils at varying elevations exhibited markedly distinct responses to increased LST. High-elevation soils demonstrated heightened sensitivity to LST regarding carbon stocks, with a significant increase in the rate of heterotrophic respiration (Rh) due to warming, while low-elevation soils displayed reduced sensitivity to temperature owing to robust microbial adaptations. Increased LST expedited the breakdown of organic matter in high-altitude soils by prolonging the duration of microbial efficacy [67].

Prior research has validated the substantial influence of NDVI on the geographical distribution of SOM [1,53]. Vegetation serves as the primary source of SOM and perpetually contributes organic matter to the soil via apoplastic inputs and root secretions [68]. Moreover, vegetation indirectly governs the dynamics of SOM by altering the soil microenvironment and affecting microbial activity [69]. This pattern has been corroborated in research across various global locations, including North America [70], Australia [71,72], Northwest China [2], and Spain [73], within diverse ecosystems.

The findings demonstrate that soil moisture is the paramount element affecting the geographical variation in SOM, with MSM exhibiting the greatest relative significance, followed by MSMS6 and MSMS3 in second and third positions, respectively. Soil moisture, as a crucial determinant of net primary productivity, directly influences the intake of SOM and indirectly governs its output by affecting soil microbial activity and decomposition rates [69]. Soil moisture has been identified as a significant environmental element influencing the geographical variability of SOM in the Gongtang Reservoir watershed in China [74], northern Ningxia [75], Changbai Mountain [76], Flanders, Belgium [77], Australia [78], and Florida, USA [79]. The relatively low importance of topographic variables (e.g., Elevation, Slope) observed in this study can be attributed to environmental homogeneity and data generalization. Firstly, the study area is a typical alluvial plain characterized by flat terrain, where the natural gradient of topographic attributes is minimal compared to hilly regions [75]. Secondly, as noted by the reviewer, the SRTM DEM (30 m) used in this study represents a generalized surface. Previous research indicates that such coarse-resolution data often fail to capture micro-topographic variations (e.g., small depressions or ridges) that regulate local soil moisture and SOM dynamics in low-relief landscapes [80]. Therefore, the limited predictive power of topography here likely reflects the inability of the input data to resolve these fine-scale micro-features rather than a lack of pedological relevance.

#### 5.1.2. Relative Importance of Time-Series NDVI Features

This study incorporated time-series NDVI features as covariates for model development. The significance of both N_Phase and N_Min exceeded that of the conventional MNDVI variables, indicating that the time-series features might more precisely represent the relationships between changes in vegetation dynamics and SOM. This finding aligns with Guo [81]. The incorporation of time-series features markedly enhanced the predictive accuracy of SOM prediction, with its operational mechanism potentially evident in the following aspects: the time-series NDVI features can proficiently encapsulate the composite productivity traits of vegetation throughout the growth cycle [82]. N_Max represents the apex of vegetation growth, while N_Min denotes the nadir of vegetation cover, typically observed during the peak season and the dormant phase in winter or under conditions of extreme drought or other stressors [83]. N_GSL measures the duration of active vegetation growth and illustrates the influence of climate on vegetation phenology [84], while N_Amplitude serves as a metric for the intensity of variations in vegetation growth, with higher values signifying more pronounced alterations in vegetation dynamics [85]. N_Phase, conversely, offers a crucial foundation for evaluating the status of plant growth by delineating the temporal characteristics of the vegetation growth cycle [86]. The characteristics of vegetation productivity directly influence organic carbon inputs, such as litter, hence regulating the dynamic equilibrium of SOM concentration. Secondly, temperature influences the climatic processes of vegetation by modulating photosynthesis efficiency, whereas alterations in precipitation directly impact the efficacy of plant’s water usage. Variations in temperature and precipitation modify the hydrothermal conditions necessary for vegetation growth, resulting in subsequent alterations in vegetation development [87]. The dynamic traits of vegetation growth fundamentally represent the spatial variability of regional hydrothermal conditions, and alterations in these conditions influence the structure and function of microbial communities, which subsequently govern changes in SOM [52].

#### 5.1.3. Significance of Multitemporal Multispectral Data

The SWIR band exhibited the highest explanatory capacity for SOM prediction among the multispectral remote sensing data. This observation aligns with the conclusions of Viscarra et al. [88], Peng et al. [89], and Gholizadeh et al. [90], confirming that SWIR bands significantly influence the prediction of surface SOM. Furthermore, Ma [91] demonstrated a substantial negative association between soil reflectance and SOM, exhibiting the most pronounced inverse correlation coefficient (*r* = −0.827) in the NIR band. The multiple regression model utilizing the inverse of the reflectance from the 4th, 5th, and 6th bands yielded favorable inverse findings (*R*^2^ = 0.86). Akbari et al. [92] conducted a comparative study indicating that the artificial neural network (ANN) model exhibited the highest prediction accuracy during the dry season (June) in arid and semiarid regions, utilizing Landsat 8 data in conjunction with various modeling approaches. The prediction model developed by Yang et al. [93] utilizing Landsat 8 data and multiple stepwise regression demonstrated a significant correlation between SOM and reflectance across all bands, thereby affirming the reliability of multi-spectral data in SOM prediction.

### 5.2. Comparative Analysis of the Applicability and Fusion Strategies of Wavelet Transform

Wavelet transform, as the fundamental tool for multiscale analysis, possesses distinct advantages in remote sensing image processing. The fundamental process involves deconstructing the original image into low-frequency approximation components and high-frequency detail components at varying resolutions by multiscale decomposition [94]. The application of remote-sensing multispectral data fusion markedly enhances spatial resolution by precisely extracting high-frequency spatial information from panchromatic images and adaptively integrating it into the multispectral data, all while preserving superior spectral characteristics [95]. The non-extractive DWT is notably exceptional in spectral fidelity due to its shift-invariant characteristic [96]. Numerous experts have consistently advocated for the advancement of wavelet transform fusion technology in the realm of methodology improvement. Li [97] introduced a multiple DWT fusion algorithm, and experimental results demonstrated its superiority over conventional HIS, PCA, and DWT techniques. Xing [98] developed a fusion rule system predicated on regional energy and standard deviation, distinguishing between low-frequency and high-frequency components. Wang [99] integrated the theory of compressed perception with the wavelet transform to enhance the fusion process of high-frequency components through the application of a region energy matching technique, resulting in substantial improvements in key metrics such as information entropy and average gradient. Patel [100] meticulously examined the impact of wavelet decomposition layers on fusion efficacy and employed Petrovic for quantitative assessment, validating that the method proficiently preserves edge characteristics while avoiding spectrum distortion. In recent years, the synergistic integration of the wavelet transform with other methodologies has demonstrated enhanced potential. Meng et al. [21] merged the RW-WDT with spectral band segmentation to effectively achieve the comprehensive fusion of several features, thereby establishing a technological framework for remote sensing modeling of soil attributes. Collectively, these research findings demonstrate that the wavelet transform remains indispensable in the domain of remote sensing information fusion due to its superior time-frequency localization properties.

To further validate the specific advantages of the proposed LEW-DWT fusion strategy, we conducted a quantitative comparison against established fusion methods. It is worth noting that while the Spatial and Temporal Adaptive Reflectance Fusion Model (STARFM) is widely used for generating synthetic imagery with high spatiotemporal resolution [12], this study aims to exploit the spectral features of real multi-temporal Landsat observations rather than filling data gaps in time series. Therefore, this study focuses on feature-level stacking rather than spatiotemporal synthesis. Consequently, we established two directly comparable baselines: (1) Simple Concatenation, where the resampled multi-source bands were directly stacked as input features, representing a baseline without mathematical transformation; and (2) Traditional DWT, which utilizes the discrete wavelet transform but employs a simple “mean rule” for fusing coefficients instead of local energy weighting. The comparative results (Table 11) reveal a distinct hierarchy in performance. The Simple Concatenation method yielded the lowest accuracy (RMSE = 1.45, R^2^ = 0.53), likely due to its inability to effectively extract multi-scale spatial features and suppress noise. Traditional DWT improved performance by incorporating frequency-domain information (RMSE = 1.37, R^2^ = 0.57). However, the proposed LEW-DWT achieved the highest accuracy (RMSE = 1.29, R^2^ = 0.62). Quantitatively, compared to Traditional DWT and Simple Concatenation, LEW-DWT reduced the RMSE by approximately 5.8% and 11.0%, respectively. This demonstrates that the local energy weighting rule effectively preserves critical spatial details and texture information from high-resolution imagery, showing a significant applicability advantage over traditional fusion or simple stacking for precise field-scale SOM mapping.

### 5.3. Model Performance

This study examines the comparative performance of the RF and CNN model in SOM prediction, revealing that CNN far surpasses RF across all evaluation metrics, aligning with findings from leading research. Nonetheless, there remains potential for enhancement in the accuracy of this study relative to leading international studies. Taghizadeh-Mehrjardi et al. [101] employed CNN and RF models to simulate the clay, sand, and silt contents of surface soil using 1524 soil samples from central Iran. The *R*^2^ for CNN model in predicting clay, sand, and silt content were 0.70, 0.73, and 0.65, respectively, while the *R*^2^ for the RF model were 0.49, 0.54, and 0.55, respectively. Furthermore, Yang et al. [56] integrated environmental factors, vegetation indices, and MODIS data into a CNN model for SOC prediction, resulting in an average *R*^2^ improvement of 24.68% over RF. This finding aligns with the present study, which observed a 16.98% enhancement in the *R*^2^ of CNN compared to RF. In the black soil region of northeastern China, Meng et al. [21] further substantiated the advantages of CNN. The study demonstrated that by integrating TSS features for SOC prediction, the CNN model achieved an *R*^2^ of 0.86, reflecting a 13.16% enhancement over the RF model’, so showcasing the CNN’s superior capability in processing remote sensing time-series data. Although the CNN model combined with LEW-DWT fusion achieved a robust accuracy (*R*^2^ = 0.62), it is notably lower than the accuracy reported by Meng et al. [21] (*R*^2^ = 0.86). This discrepancy is primarily attributed to differences in geo-environmental characteristics and data availability, rather than the capability of the CNN model itself. Firstly, Meng et al.’s study focused on the Black Soil region of Northeast China, characterized by high SOM content and strong spectral distinctiveness, whereas our study area (Yucheng) is located in the Yellow River alluvial plain with low SOM content (mean 20.12 g·kg^−1^) and a narrow variation range. Secondly, and crucially, Meng et al. incorporated GF-5 hyperspectral data, which contains hundreds of continuous spectral bands capable of capturing subtle SOM absorption features. In contrast, our study relies on Landsat 8 multispectral imagery. While multispectral data has lower spectral resolution, it offers advantages in terms of free accessibility and high temporal frequency. Achieving an *R*^2^ of 0.62 using only multispectral data demonstrates that our proposed LEW-DWT strategy effectively compensates for the lack of spectral bands by enhancing spatial texture features, providing a cost-effective solution for regional monitoring. Zhang et al. [82] developed a CNN-LSTM model in Xuancheng city to extract spatial aspects of static environmental variables and temporal dynamic features of the 10-year vegetation index, resulting in a 4.72% enhancement in *R*^2^ compared to the RF model. Comparatively speaking, our LEW-DWT + CNN framework possesses unique advantages in enhancing spatial features. While CNN-LSTM focuses on the trajectory of vegetation changes, our method prioritizes the integration of multi-temporal spectral details to sharpen spatial textures. For fragmented agricultural landscapes like Yucheng, enhancing spatial resolution and texture through LEW-DWT has proven to be an efficient strategy for distinguishing field-scale SOM variations. The outcomes of this work align with existing literature on prediction accuracy and further substantiate the applicability of deep learning models in soil attribute prediction.

CNNs can represent nonlinear interactions due to their distinctive architectural design, resulting in effective learning capabilities while handling complicated, high-volume input data [102], so rendering them especially adept at processing multiband remote sensing images. In this work, CNNs provide enhanced predictive performance compared to traditional algorithms, a conclusion that supports the results of Yang [55] and Wadoux [56]. Nonetheless, CNNs possess certain constraints in their applications. Constructing dependable CNN models often depends on extensive training datasets [103]. CNNs necessitate meticulous fine-tuning and careful consideration of overfitting during the training process [104].

## 6. Limitations and Outlook

Despite the accomplishments of this study in SOM prediction and mapping, certain limitations persist that require additional optimization in subsequent research. (1) The Landsat 8 imagery utilized covers the years 2014 to 2023; however, cloud interference resulted in the absence of suitable imagery for certain years (e.g., 2016, 2021, 2022) during the bare soil period, potentially impacting the completeness of the time series data. (2) This study utilized a dataset of 198 samples to train the CNN model. While this sample size is relatively small for typical deep learning applications, we mitigated the risk of overfitting by optimizing the model architecture. Specifically, we employed a shallow, lightweight CNN structure (comprising only three convolutional layers) to limit the total number of trainable parameters and integrated Dropout layers (rates of 0.1–0.2) to enhance generalization. The 10-fold cross-validation results demonstrated a narrow gap between training and validation errors, along with a low standard deviation across folds, indicating satisfactory model stability. Nevertheless, the risk of overfitting cannot be entirely eliminated. Future research should focus on expanding the soil spectral library to further validate the model’s applicability across larger geographical scales and more diverse soil types. (3) The limited spatial resolution (250 m) of MODIS data may insufficiently detect nuanced variations at the agricultural scale. (4) Anthropogenic elements, such as road network density and built-up volume, may inadequately represent the effects of modern agricultural management methods due to the irregular temporal spans of the data (2010–2022). (5) The model shown strong performance on the cultivated area of Yucheng city; nevertheless, it has not been validated across various soil types, necessitating further assessment of its generalizability. (6) Finally, the scale mismatch inherent in fusing multisource data warrants discussion. In this study, coarse-resolution environmental covariates (e.g., climate and soil moisture data at 1–5 km) were resampled to 30 m to align with the Landsat imagery. While this is a common strategy in digital soil mapping to incorporate regional environmental trends, it does not strictly increase the information content of the coarse variables. Correlating point-scale soil samples with resampled coarse pixels—which represent averages over large areas—ignores intra-pixel spatial heterogeneity and may introduce “change of support” errors. Although this approach was necessary to preserve the high-resolution local details from Landsat data required for precision agriculture, future studies should explore advanced multi-scale modeling or area-to-point kriging techniques to better mitigate these scaling uncertainties.

Future study may investigate the amalgamation of higher geographical and temporal resolution data (e.g., Sentinel-2, GF series satellites) with UAV remote sensing to enhance field-scale information. Spatiotemporal fusion models, such as Con-vLSTM or transformers, should be employed to improve the capacity for capturing long-term temporal features.

## 7. Conclusions

This study predicted and mapped cropland SOM in Yucheng City by proposing a Local Energy Weighted Discrete Wavelet Transform (LEW-DWT) method to fuse multi-temporal Landsat 8 images, combined with time-series NDVI features and environmental variables. We constructed and compared Random Forest (RF) and Convolutional Neural Network (CNN) models to evaluate the effectiveness of this multisource fusion framework. The main conclusions are as follows:

(1) Advantages of LEW-DWT fusion strategy: The proposed LEW-DWT method demonstrated superior performance in preserving critical spatial details and texture information compared to traditional fusion methods. Quantitative comparisons indicated that LEW-DWT achieved higher spatial fidelity (lower SAM, higher IE and AG) and reduced the prediction RMSE by approximately 5.8% and 11.0% compared to Traditional DWT and Simple Splicing, respectively. The combination of seven Landsat 8 images fused via LEW-DWT provided the optimal multispectral dataset (*R*^2^ = 0.49), confirming its ability to capture dynamic soil variations in fragmented agricultural landscapes where simple resampling often fails.
(2) Dominant Environmental Drivers: Based on the interpretable RF model analysis, soil moisture (specifically MSM, MSMS6, and MSMS3) was identified as the primary environmental variable controlling SOM distribution, contributing 45.84% of the total importance. This underscores the critical regulatory function of moisture conditions in SOM accumulation and decomposition. Among remote sensing features, the NDVI Phase (representing vegetation phenology) and the SWIR1 band were the most significant predictors, validating the necessity of integrating multi-temporal spectral and phenological information.
(3) Superiority and Robustness of the Optimized CNN: The CNN model consistently outperformed the RF model across all input combinations. Using the composite Ev–Tn–Mm features, the CNN achieved the highest accuracy(*R*^2^ = 0.62, RMSE = 1.29). Crucially, despite the limited sample size (*N* = 198), the adoption of a shallow network architecture with smaller filters (3 × 3) effectively mitigated overfitting while successfully extracting local spatial patterns and non-linear relationships. This confirms that a properly designed CNN is highly effective for regional SOM mapping even with restricted field data. The resulting map shows SOM concentrations ranging from 14.49 to 28.62 g/kg, providing high-precision data support for precision agriculture and soil carbon management in Yucheng City.

## Figures and Tables

**Table 1:** Landsat 8 OLI image information.

Image Type	ID	Acquisition Date	Image Type	ID	Acquisition Date
Landsat8 OLI	1	6 October 2014	Landsat8 OLI	5	27 September 2019
	2	9 October 2015		6	22 October 2020
	3	28 September 2017		7	15 October 2023
	4	1 October 2018			

**Table 2:** Time-series of the NDVI data.

Data Type	ID	Start Time	End Time	Data Type	ID	Start Time	End Time
MODIS13Q1	1	1 January 2014	19 December 2014	MODIS13Q1	6	1 January 2019	19 December 2019
	2	1 January 2015	19 December 2015		7	1 January 2020	18 December 2020
	3	1 January 2016	18 December 2016		8	1 January 2021	19 December 2021
	4	1 January 2017	19 December 2017		9	1 January 2022	19 December 2022
	5	1 January 2018	19 December 2018		10	1 January 2023	19 December 2023

**Table 3:** Environmental covariates used for SOM prediction.

Typology	Name	Abbreviation	Resolution	Data Sources	Time Span
Climate	Mean annual air temperature	MAAT	1 km	WorldClim version 2	1970–2000
	Mean diurnal range	MDR	1 km		
	Mean annual range	MAR	1 km		
	Air temperature seasonality	ATS	1 km		
	Maximum air temperature of warmest month	MAWM	1 km		
	Minimum air temperature of coldest month	MACM	1 km		
	Mean annual solar radiation	MASR	1 km		
	Wind speed	WS	1 km		
	Water vapor pressure	WVP	1 km		
	Mean annual precipitation	MAP	1 km	Science Data Bank	1960–2020
	Precipitation standard deviation	PSD	1 km		
Parent materials	Regolith thickness	RT	100 m	[44]	-
	Rock lithology	RL	250 m	U.S. Geological Survey (USGS)	-
Topographic	Elevation	DEM	250 m	USGS DEM	-
	Slope gradient	SG	250 m	Processed from Elevation data	-
	Slope aspect	SA	250 m		-
	Planform curvature	PLC	250 m		-
	Profile curvature	PRC	250 m		-
	Topographic wetness index	TWI	250 m		-
	Topographic position index	TPI	250 m		-
Vegetation	Mean normalized difference vegetation index	MNDVI	500 m	USGS MOD13Q1	2004–2023
	Standard deviation of normalized difference vegetation index	SDNDVI	500 m		
Land surface thermal	Highest daytime Land surface temperature (LST)	HDLST	1 km	Resources and Environmental Science Data Platform	2004–2023
	Mean daytime LST	MDLST	1 km		
	Standard deviation of daytime LST	SDDLST	1 km		
	Highest nighttime LST	HNLST	1 km		
	Mean nighttime LST	MNLST	1 km		
	Standard deviation of nighttime LST	SDNLST	1 km		
	Mean daytime and nighttime LST in spring (March, April, May)	MLSTS3	1 km		
	Mean daytime and nighttime LST in summer (June, July, August)	MLSTS6	1 km		
	Mean daytime and nighttime LST in autumn (September, October, November)	MLSTA	1 km		
	Mean daytime and nighttime LST in winter (December, January, February)	MLSTW	1 km		
Soil	Groundwater table depth	GWTD	1 km	[45]	-
	Mean soil moisture	MSM	5 km	[43]	2003–2018
	Standard deviation of soil moisture	SDSM	5 km		
	Highest soil moisture	HSM	5 km	Processed from MSM data	2003–2018
	Lowest soil moisture	LSM	5 km		
	Mean soil moisture in spring (March, April, May)	MSMS3	5 km		
	Mean soil moisture in summer (June, July, August)	MSMS6	5 km		
	Mean soil moisture in autumn (September, October, November)	MSMA	5 km		
	Mean soil moisture in winter (December, January, February)	MSMW	5 km		
Anthropogenic variables	Population density	PD	1 km	Resources and Environmental Science Data Platform	2019
	Built-up volume	BUV	100 m	GHSL-Global Human Settlement Layer	2010
	Road network density	RD	1 km	Global Change Research Data Publishing & Repository	2022
	Hourly anthropogenic heat flux	HAHF	1 km	[46]	2010

**Table 4:** Number of multitemporal multispectral images after permutation and combination.

CN	NRC	Abbreviation	CN	NRC	Abbreviation
1	1	MMI_1_	5	15	MMI_5_
2	6	MMI_2_	6	6	MMI_6_
3	15	MMI_3_	7	1	MMI_7_
4	20	MMI_4_			

Note: CN represents the number of images considered; NRC represents the number of results after permutation.

**Table 5:** Combinations of different input variables.

ID	Input	Covariates
1	Ev	Environment variables
2	Ev-Tn-Mm	Environment variables + Time-series NDVI characteristics + Multitemporal multispectral images

**Table 6:** Detailed information of the CNN architecture.

CNN Layer Type	Filter Size	Number of Filters/Neurons	Activation Function
Convolutional layer	3 × 3	32	ReLU
Maximum pooling layer	2 × 2	-	-
Convolutional layer	2 × 2	48	ReLU
Dropout layer (0.2)	-	-	-
Convolutional layer	2 × 2	24	ReLU
Convolutional layer	2 × 2	12	ReLU
Dropout layer (0.2)	-	-	-
Flatten layer			
Fully connection layer	-	80	ReLU
Dropout layer (0.1)	-	-	-
Fully connection layer	-	15	ReLU
Output layer	-	1	Linear

**Table 7:** SOM statistics of the sampling points in the study area.

SOM Dataset	Sample Size (*N*)	Maximum Value (g·kg^−1^)	Mean Value (g·kg^−1^)	Minimum Value (g·kg^−1^)	Standard Deviation Value	Coefficient of Variation (%)
Total sample size	198	27.65	20.12	14.73	2.42	12

**Table 8:** Maximum accuracy averages for different numbers of multitemporal multispectral images and the date the fused image was taken.

NRC	Date	MAE	RMSE	*R* ^2^	Abbreviation
1	2020	1.35	1.70	0.41	MMI_1_
2	2015, 2020	1.31	1.69	0.43	MMI_2_ *
3	2014, 2017, 2020	1.30	1.68	0.43	MMI_3_ *
4	2015, 2017, 2019, 2020	1.29	1.67	0.44	MMI_4_ *
5	2014, 2015, 2017, 2020, 2023	1.29	1.65	0.45	MMI_5_ *
6	2014, 2015, 2017, 2018, 2020, 2023	1.28	1.64	0.46	MMI_6_ *
7	2014, 2015, 2017, 2018, 2019, 2020, 2023	1.26	1.60	0.49	MMI_7_

Note: NRC denotes the number of input images; Date denotes the time when the image was taken; * denotes the best data fusion image for SOM content prediction with the number of input multitemporal multispectral images.

**Table 9:** Quantitative evaluation of fusion quality for different fusion methods.

Method	SAM (Rad)	Entropy (IE)	Average Gradient (AG)
Traditional DWT	0.056	5.12	3.85
LEW-DWT	0.038	5.65	4.92

**Table 10:** Indicators of the predictive accuracy of different predictive models.

Model	Input	MAE	RMSE	*R* ^2^
RF	Env	1.28	1.59	0.47
	Env + MMI + TNc	1.18	1.52	0.53
CNN	Env	1.13	1.50	0.54
	Env + MMI + TNc	0.91	1.29	0.62

**Table 11:** Comparison of prediction accuracy among different fusion strategies using the CNN model.

Fusion Strategy	Description	MAE	RMSE	*R* ^2^
Simple Splicing	Direct concatenation of bands	1.12	1.45	0.53
Traditional DWT	DWT with Mean-rule fusion	1.02	1.37	0.57
LEW-DWT	DWT with Local Energy weighting	0.91	1.29	0.62

## Data Availability

The sources of all the data have been provided in the article; if they are still needed, please contact the corresponding author to obtain them.
